# Knowledge, attitudes and practices related to stroke in Ghana and Nigeria: A SIREN call to action

**DOI:** 10.1371/journal.pone.0206548

**Published:** 2018-11-16

**Authors:** Carolyn Jenkins, Bruce Ovbiagele, Oyedunni Arulogun, Arti Singh, Benedict Calys-Tagoe, Rufus Akinyemi, Aliyu Mande, Ezinne Sylvia Melikam, Albert Akpalu, Kolawole Wahab, Fred Stephen Sarfo, Taofeeq Sanni, Godwin Osaigbovo, Hemant K. Tiwari, Reginald Obiako, Vincent Shidali, Philip Ibinaiye, Josephine Akpalu, Godwin Ogbole, Lukman Owolabi, Ezinne Uvere, Raelle Taggae, Abiodun Moshood Adeoye, Mulugeta Gebregziabher, Adeseye Akintunde, Oladimeji Adebayo, Ayodipupo Oguntade, Ayotunde Bisi, Kenneth Ohagwu, Ruth Laryea, Peter Olowoniyi, Isah Suleiman Yahaya, Samuel Olowookere, Frederick Adeyemi, Morenikeji Komolafe, Michael Bimbola Fawale, Taofiki Sunmonu, Ugochukwu Onyeonoro, Lucius Chidiebere Imoh, Wisdom Oguike, Taiye Olunuga, Phillip Kolo, Okechukwu S. Ogah, Richard Efidi, Ijezie Chukwuonye, Andrew Bock-Oruma, Dorcas Owusu, Chidi Joseph Odo, Moyinoluwalogo Faniyan, Osimhiarherhuo Adeleye Ohnifeman, Olabanji Ajose, Luqman Ogunjimi, Shelia Johnson, Amusa Ganiyu, Paul Olowoyo, Adekunle Gregory Fakunle, Afolaranmi Tolulope, Temitope Farombi, Monica Oghome Obiabo, Mayowa Owolabi

**Affiliations:** 1 College of Nursing, Medical University of South Carolina, Charleston, South Carolina, United States of America; 2 Neurology, College of Medicine, Medical University of South Carolina, Charleston, South Carolina, United States of America; 3 University College Hospital and University of Ibadan, Ibadan, Nigeria; 4 Kwame Nkrumah University of Science and Technology, Kumasi, Ghana; 5 University of Ghana Medical School, Accra, Ghana; 6 Federal Medical Center, University of Ibadan, Abeokuta, Nigeria; 7 Aminu Kano Teaching Hospital, Kano, Nigeria; 8 University of Ilorin Teaching Hospital, Ilorin, Nigeria; 9 Komfo Anokye Teaching Hospital, Kumasi, Ghana; 10 Community Medicine, Federal Teaching Hospital, Ido-Ekiti, Nigeria; 11 Jos University Teaching Hospital, Jos, Nigeria; 12 Biostatistics, University of Alabama, Birmingham, Alabama, United States of America; 13 Ahmadu Bello University Teaching Hospital, Zaria, Nigeria; 14 Public Health Sciences, College of Medicine, Medical University of South Carolina, Charleston, South Carolina, United States of America; 15 Ladoke Akintola University of Technology & Teaching Hospital, Ogbomoso, Nigeria; 16 Federal Medical Center, Umuahia, Nigeria; 17 Bayero University/Aminu Kano Teaching Hospital, Kano, Nigeria; 18 Obafemi Awolowo University Teaching Hospitals, Ile-Ife, Nigeria; 19 Federal Medical Center, Owo, Nigeria; 20 Pathology, Jos University Teaching Hospital, Jos, Nigeria; 21 Medicine, University of Ilorin, University of Ilorin Teaching Hospital Ilorin, Nigeria; 22 Radiology, College of Medicine, University College Hospital, Ibadan, Nigeria; 23 Internal Medicine, Federal Medical Centre, Umuahia, Nigeria; 24 Delta State University Teaching Hospital, Oghara, Nigeria; 25 Cardiology, Jos University Teaching Hospital, Jos, Nigeria; 26 Neurology, Medicine, Federal Teaching Hospital, Ido-Ekiti College of Medicine and Health Sciences, Afe Babalola University, Ado-Ekiti, Nigeria; 27 Environmental Health Sciences, Public Health, University of Ibadan, Ibadan, Nigeria; 28 Community Medicine, University of Jos/Jos University Teaching Hospital, Jos, Nigeria; 29 Neurology Unit, Chief Tony Anenih Geriatric Center, University College Hospital, Ibadan, Nigeria; 30 Internal Medicine, Delta State University Teaching Hospital, Oghara, Nigeria; ESIC Medical College & PGIMSR, INDIA

## Abstract

**Introduction:**

Stroke is a prominent cause of death, disability, and dementia in sub-Saharan Africa (SSA). The Stroke Investigative Research and Education Network works collaboratively with stroke survivors and individuals serving as community controls to comprehensively characterize the genomic, sociocultural, economic and behavioral risk factors for stroke in SSA.

**Purpose:**

In this paper, we aim to: i) explore the attitudes, beliefs, and practices related to stroke in Ghana and Nigeria using the process of qualitative description; and ii) propose actions for future research and community-based participation and education.

**Methods:**

Stroke survivors, their caregivers, health care professionals, and community representatives and faith-based leaders participated in one of twenty-six focus groups, which qualitatively explored community beliefs, attitudes and practices related to stroke in Ghana and Nigeria. Arthur Kleinman’s Explanatory Model of Illness and the Social Ecological Model guided the questions and/or thematic analysis of the qualitative data. We hereby describe our focus group methods and analyses of qualitative data, as well as the findings and suggestions for improving stroke outcomes.

**Results and discussion:**

The major findings illustrate the fears, causes, chief problems, treatment, and recommendations related to stroke through the views of the participants, as well as recommendations for working effectively with the SIREN communities. Findings are compared to SIREN quantitative data and other qualitative studies in Africa. As far as we are aware, this is the first paper to qualitatively explore and contrast community beliefs, attitudes, and practices among stroke survivors and their caregivers, community and faith-based leaders, and health professionals in multiple communities within Nigeria and Ghana.

## Introduction

Stroke is a leading cause of death and disability globally [1] and the incidence of stroke is increasing in Low-and Middle-Income Countries (LMIC) particularly in Africa [2–6]. Rates are decreasing in high income countries (HIC); however, in Africa, the rates are high, increasing, disproportionally afflicting young and middle-aged adults, and disability and death are much worse than in HIC [7–12].

The Stroke Investigative Research and Education Network (SIREN) seeks to comprehensively characterize the genomic, sociocultural, economic and behavioral risk factors leading to the development of clinical risk factors for stroke and sub-clinical disease which over time may result in clinically apparent stroke [13–17]. An earlier publication [14] presented an overview of eight study sites located in six cities, six in Nigeria and two in Ghana, and the protocol for community engagement and research across these sites. Other sites have been added to SIREN and the added sites did not collect focus group (FG) data for this study, but the team of investigators worked together to translate, analyze and interpret the data and prepare the manuscript. It is important to note that this genomic research does not involve an intervention that focuses on immediate improvement of stroke outcomes; SIREN focuses on recruiting 3000 case-control pairs to identify particular i) genetic (relating to genes, genetic variation, and heredity); ii) genomic (relating to recombinant DNA, DNA sequencing methods, and bioinformatics to sequence, assemble, and analyze the function and structure of the complete set of DNA within a single cell); and iii) phenomic (relating to physical and biochemical traits of persons—as they change in response to genetic mutation and environmental influences) factors related to causation or prevention of stroke.

As a member of the H3Africa Initiative, the SIREN team adheres to the High-Level Principles on Ethics, Governance and Resource Sharing which state, *“Research networks and programs should incorporate appropriate engagement with the communities participating in the research as an integral element* [18].*”* SIREN researchers connect with people in the community to learn from them, in order to identify and work effectively with surrounding communities, not only to recruit research participants for SIREN, but also to design interventions that could be tested in future trials to reduce the tremendous burden of stroke in sub-Sahara Africa (SSA). A local advisory board of community leaders in each of the cities where SIREN sites are located, guides the program’s work within that community.

### Purpose

The purpose of this paper is to report the qualitative findings [19, 20], from FGs that explored the knowledge, attitudes and practices related to stroke, stroke risk factors, genetic testing and genomics among persons who had a stroke and their caregivers, as well as key community constituents including faith-based leaders and health care workers.

## Methods

To facilitate comprehensive reporting of the processes and results, recommendations from Consolidated Criteria for Reporting Qualitative Research (COREQ): a 32-item checklist for interviews and FGs [21]; the Standards for Reporting Qualitative Research: a Synthesis of Best Practices [22]; and the Evolving Guidelines for Publishing Qualitative Research Studies in Psychology and Related Fields [23] all served as guides for development of this manuscript. COREQ suggests presenting the research in three domains: research team and reflexivity; study design; and analysis and findings [21].

### Domain 1: Reflexivity [24] and bias in SIREN research team

The SIREN team includes members who are researchers and clinicians, nurses, medical laboratory scientists, health educators, public health professionals, radiologists who handle the imaging interpretations for diagnosing stroke and its subtypes, epidemiologists, statisticians and community members in Ghana, Nigeria and the United States of America (US). The overall principal investigators for SIREN, as well as the local site principal investigators are neurologists or other physician researchers who also provide clinical care to stroke patients. The team for community engagement across sites consists of the investigators and staff with expertise in both qualitative and quantitative research as well as community engagement. All FG facilitators completed some form of tertiary education—MPH, BSc or some form of Social Science degree in Humanities. The majority have prior research experience: hospital-based, community-based or field work. Each is knowledgeable about the culture in their respective communities in Ghana and Nigeria.

Additionally, researchers at the Medical University of South Carolina (MUSC) in the USA are also part of the SIREN team. The team communicates via Skype at least 4 times per month. The community engagement investigator at MUSC and lead author of this article has: i) a long history of working with southern African American communities in the USA in community-based participatory research related to diabetes and qualitative research, but only had worked with stroke patients who also had diabetes before working with SIREN; ii) coordinated the development of the standard operating procedures for community engagement with the SIREN team; iii) developed the initial set of questions for SIREN FGs; iv) provided additional training related to FG processes; v) analyzed and coordinated discussion of the FG data with the SIREN team; and vi) prepared the manuscript over 2–3 years in collaboration with the SIREN team.

Ghanaian and Nigerian colleagues: i) provided training and education for the MUSC community engagement investigator about the culture in Ghana and Nigeria; ii) refined the FG questions; iii) conducted all FGs; iv) coordinated the translation process; v) provided input into their reflexivity and biases; vi) provided data related to ‘member checking;’ and vii) input into the development of the manuscript. All SIREN investigators continue to work together via Skype teleconferencing each month. Trained investigators and staff (including both males and females) who spoke the language or dialect and moderated the FGs at each site had previously worked with or knew some of the FG participants. All investigators have personal interests in stroke and genomics or phenomics and most are physician researchers or public health leaders in their respective sites with prior work with stroke. Although all FG facilitators-moderators, and note takers had previously conducted FG sessions, they participated in training using the SIREN CE core protocol to help ensure uniformity of practices and replicability of methods.

Although our team has discussed bias, which we define as predisposition(s) or preconceived opinion(s) that prevent team members from impartially evaluating the “facts” that have been presented by the FG participants, bias has been somewhat difficult to identify! Several team members have stated that they are first generation college graduates who have experienced poverty and unhealthy lifestyles and several have family histories of stroke and deaths from stroke. However, the team’s education and awareness of the scientific literature related to stroke and risk factors, and the interests in the genomics and phenomics of stroke certainly may introduce bias in interpreting the information presented. Thus, we have worked together to create a very diverse group of clinicians and researchers, as well as feedback from community members, to focus on interpreting the data and creating a high level of agreement related to data interpretation. The team has discussed and interpreted the findings, verified findings and worked together to create transparency in reporting the findings by identifying and reporting common themes and using direct quotes from the participants to illustrate the common themes. Additionally, we use theoretical models and methods that have been developed and tested by other researchers and communities to help develop a framework for study design, data collection, analysis and reporting. We also recognize the uniqueness of our research and the selected models allow us to report our unique findings. We triangulate our findings with those from our SIREN survey and other quantitative data.

### Domain 2: Study design

The literature describing the demographics of the study sites and the FG has been previously discussed in a prior publication The rationale for using FGs to explore attitudes, beliefs, and practices related to stroke in Ghana and Nigeria allows the SIREN team to capture qualitative data that may be missed with our SIREN quantitative survey. Also, the survey data were collected from stroke survivors and control subjects from the community, while the FGs examined attitudes, beliefs and practices from stroke survivors, family, community and faith-based leaders and health professionals. Also, both FGs and surveys were conducted during the same period of time (although ideally if the focus groups were conducted prior to the development of the survey, the findings from the FGs could have been further explored with larger numbers of person in the survey). Study sites that assisted with the FG study design and then participated in the FG research included two sites in Northern Nigeria (Kano and Zaria), four sites in Southern Nigeria (two in Abeokuta and two in Ibadan), and two in Ghana (Accra and Kumasi). Each of the sites planned 3–4 FG sessions including one to two FGs with stroke survivors and their caregivers, one with community and faith-based leaders, and one with health care providers and researchers. To standardize the FG process, materials and training were provided for FG discussions, in-depth interview guides, and forms for informed consents, demographics, and note-taking. Skype trainings were provided to all FG leaders. FG methods were based on methodology developed by Kruger and Casey [25] and in-depth interview methodology developed by Tremblay [26]. The research was approved by the Ethics Committees of each site and also by the Institutional Review Board of the Medical University of South Carolina.

#### Theoretical models

Theoretical frameworks that guided our FG questions and processes across the SIREN communities were the Social Ecological Model (SEM) [27] and Kleinman’s Explanatory Model of Illness [28]. The constructs of the SEM were used to: i) explore individual and population health related to stroke including individual factors (e.g., genetics, pathophysiologic pathways, individual knowledge, beliefs, behaviors); ii) interpersonal factors (e.g., family, friends, peers, and other social relationships); iii) institutional processes (e.g., social networks, social support systems); iv) community factors (e.g., relationships among networks, health care access, living conditions); and v) public policy (e.g., local, state, and national laws/policies) [14]. Kleinman’s model [28] provided the framework for further developing the FG questions and exploring beliefs and attitudes related to stroke and stroke research [14].

#### Sampling and sample recruitment

Participants were selected through purposive and snowball sampling techniques and then recruited by SIREN investigators and staff, largely through face to face contact (n = 4 sites), telephone calls (n = 1 site) or a combination of telephone and face to face contacts (n = 2 sites). Only 6 persons refused to participate and the reasons for all were related to conflicts of time. An overview of the FGs and number of participants in each group by site is shown in Table 1. The site PIs and the community engagement coordinators used purposive sampling for recruiting both males and females, an age range representing the population in each of the 3 groups (stroke survivors, community leaders and health care providers), and persons residing in rural and urban communities. The snowball sampling techniques were used by all members of the SIREN team and the community advisory board and included recruiting hospital stroke survivors who were being discharged from the hospital, and asking those who volunteered to help recruit other stroke survivors and community leaders to participate in SIREN FGs and other SIREN activities. The community leaders were asked to recruit both stroke survivors from their communities as well as community leaders from other communities. Additionally, FG participants were recruited by the researchers during community events.

**Table 1:** SIREN focus group participant demographics.

	# of participants in each group		Age of Group	
Sites	# Females	# Males	Average or mean age of group (years)	Age range of group (years)
Ghana: Accra (University of Ghana, Korle BU Teaching Hospital)				
• Stroke Survivors	6	0	59.5	32–80
• Stroke Survivors	0	7	47.6	41–63
• Community Leaders	5	3	42.1	22–63
• Health Care Workers	5	2	41.1	26–64
Ghana: Kumasi (Kwame Nkrumah University, Komfo Anokye Teaching Hospital)				
• Stroke survivors	0	5	48.8	34–69
• Stroke survivors	6	0	57.2	45–78
• Community leaders	3	4	53.4	42–64
• Health Care Workers	3	4	50.4	43–54
Nigeria: Abeokuta (Federal Medical Centre)				
• Stroke Survivors	2	4	57.67	34–72
• Community Leaders	1	5	57.33	36–77
• Health Care Workers	4	2	38.17	31–49
Nigeria: Abeokuta (Sacred Heart Hospital)				
• Stroke survivors	0	6	52.17	38–67
• Community leaders	2	4	56.33	41–70
• Health Care Workers	5	1	36.67	29–50
Nigeria: Ibadan (Blossom Specialist Medical Center)				
• Stroke Survivors	2	4	52.3	45–60
• Community Leaders	3	3	60.2	40–85
• Health Care Workers	1	5	34.2	23–35
Nigeria: Ibadan (University of Ibadan, University College Hospital)				
• Stroke survivors	1	3	65.5	60–73
• Community leaders	1	5	52.83	40–69
• Health Care Workers	2	4	37.67	28–59
Nigeria: Kano (Bayero University, Aminu Kano Teaching Hospital)				
• Stroke Survivors	3	3	44	35–53
• Community Leaders	0	6	50	40–60
• Urban Health Care workers	2	4	35	30–45
• Rural Health Care Workers	3	3	35	30–45
Nigeria: Zaria (Ahmadu Bello University, Ahmadu Bello Teaching Hospital)				
• Stroke Survivors	2	4	53	45–60
• Community Leaders	0	6	54.5	50–60
• Health Care Workers	2	4	34	30–40
**SUB-TOTAL** **Stroke Survivors** N = 9 Groups, 58 participants **Community Leaders** N = 8 Groups, 51 participants **Health Care Workers** N = 9 Groups, 56 participants	22 15 27	36 36 29		32–80 22–85 23–64
**TOTAL N = 165 persons**	**64**	**101**		**22–85**

A total of 165 persons in 26 FG across 8 communities or health care organizations were included. Female participation was lower than males in all groups. Although this may reflect the culture of the communities, it is important to note that in Nigeria and Ghana, more males than females are hospitalized with stroke, more males are physicians and researchers, and more males are community and religious leaders. Thus, the participant gender is reflective of each of the groups. The rationale for the large number of FGs was that each of the communities are sites for SIREN recruitment in the large case-control study and each site needed to better understand the knowledge, attitudes and beliefs of their community leaders, health care providers as well as persons who had experienced a stroke and their care providers.

#### Settings

The settings for the FGs varied but most were in clinical sites. All FGs (n = 9) with stroke survivors who had been previously discharged from the hospital with a diagnosis of stroke) and their caregivers were conducted in hospital outpatient clinics. All but one of the health care providers FGs were conducted in the hospital outpatient clinics across the SIREN sites (n = 8) while one health care providers FG was conducted in a rural clinic with no associated hospital (n = 1). The community leaders FGs (n = 8) were divided between community (n = 4) and hospital outpatient clinic (n = 4) sites. Only the FG leader and the note taker were present with the participants during each of the FGs.

#### Data collection

**Fig 1:**
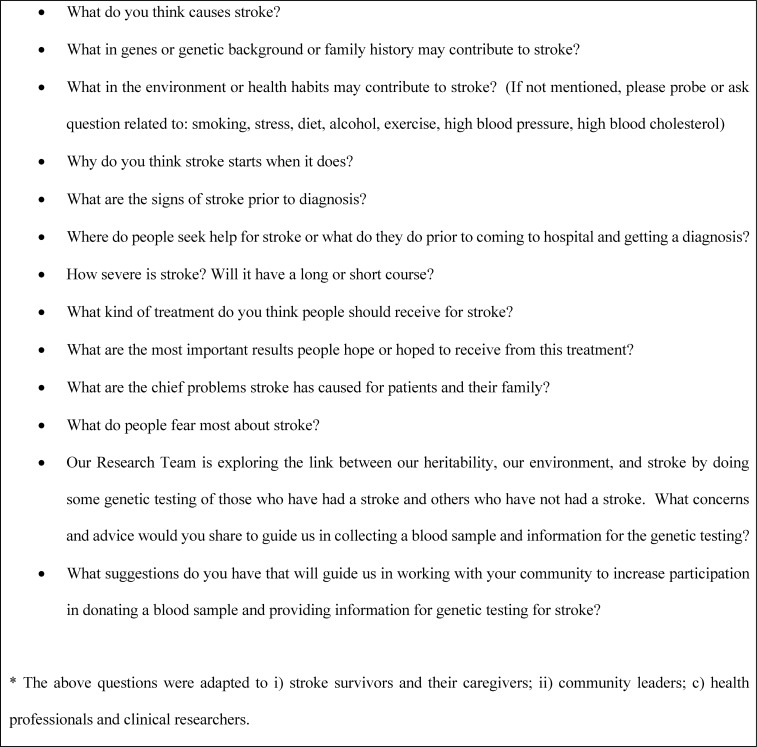
Focus group questions.

An interview guide was developed collaboratively by the SIREN community engagement investigators and collectively reviewed with all investigators and staff through teleconference meetings on Skype. An overview of the key questions is shown in Fig 1.

The site principal investigators, co-investigators, and community engagement coordinators reviewed, edited, and worked with community members to translate and back translate into the language/dialect in each of the SIREN communities. If not administered in English, the informed consent, the FG, and associated materials were administered in one of the major languages as appropriate for the participants including Hausa, Yoruba, Igbo in Nigeria and Asante Twi in Kumasi, Ghana and Ga in Accra, Ghana. The duration of the FGs ranged from 60 to 90 minutes. Credibility of the data was enhanced through summarizing and debriefing at the end of the groups. All FGs were audio digitally recorded, field notes were made during the interview, and the digital recordings were transcribed verbatim. The multiple languages and dialects in the SIREN communities presented translation challenges for the team. However, the process developed by Beaton and colleagues [29, 30] provided a guide for translation of both the FG questions into the language or dialect as well as translation of the FG results into English. Following translation into English for those FGs administered in another language, review and editing of all transcripts at each site, the digital recordings, verbatim translations, and field notes were loaded into REDCap, a secure data management and storage system at the Medical University of South Carolina for documentation. All personal identifiers were removed from the transcripts that were used for analyses. Only three of the FG transcripts were returned to a sample of participants for review prior to analysis. No edits were made by the participants.

### Domain 3: Analysis

The guiding methodological approach selected for identifying, analyzing, and presenting the data is thematic analysis [31] using the structure from the SEM [27] and Kleinman’s framework [28] for generating the initial analytic approach and codes. This combination of approaches adds to the literature by illustrating the usefulness of the approach for assessing, developing, and refining data that can be used for planning and testing interventions to decrease the tremendous burden of stroke in SSA. Thematic analysis, as an independent qualitative descriptive approach, is mainly described as “a method for identifying, analyzing and reporting patterns (themes) within data [32].”

The data were coded by the primary author (CJ) and reviewed by all members of the SIREN Community Engagement team through monthly meetings and then with the Principal Investigators (MO and BO) and the SIREN team. Major themes or nodes were first identified using the domains from Kleinman’s Explanatory Model [28]. The major themes from Kleinman [28] were then stratified by the major themes (individual including pathophysiologic pathways, genetic, constitutional, and individual risk factors; interpersonal/social relationships and living conditions; organizations and communities, and social and economic policy) of the SEM [27]. Transcripts were reviewed for themes through a continuous process of data segment comparison based on the qualitative research techniques described by Huberman and Miles [33] and Patton [34]. A codebook was developed defining themes, and a numeric theme code was assigned to each particular category of text responses. Participants’ responses were coded and sorted accordingly into differing categories. Microsoft Word was used to create tables sorted on theme code [35]. An iterative process of reading and rereading the data was used to refine the categories and ensure the coded responses fit well into the categories. The analysis was systematic and involved categorizing data into the two theoretical frameworks [27, 28].

**Fig 2:**
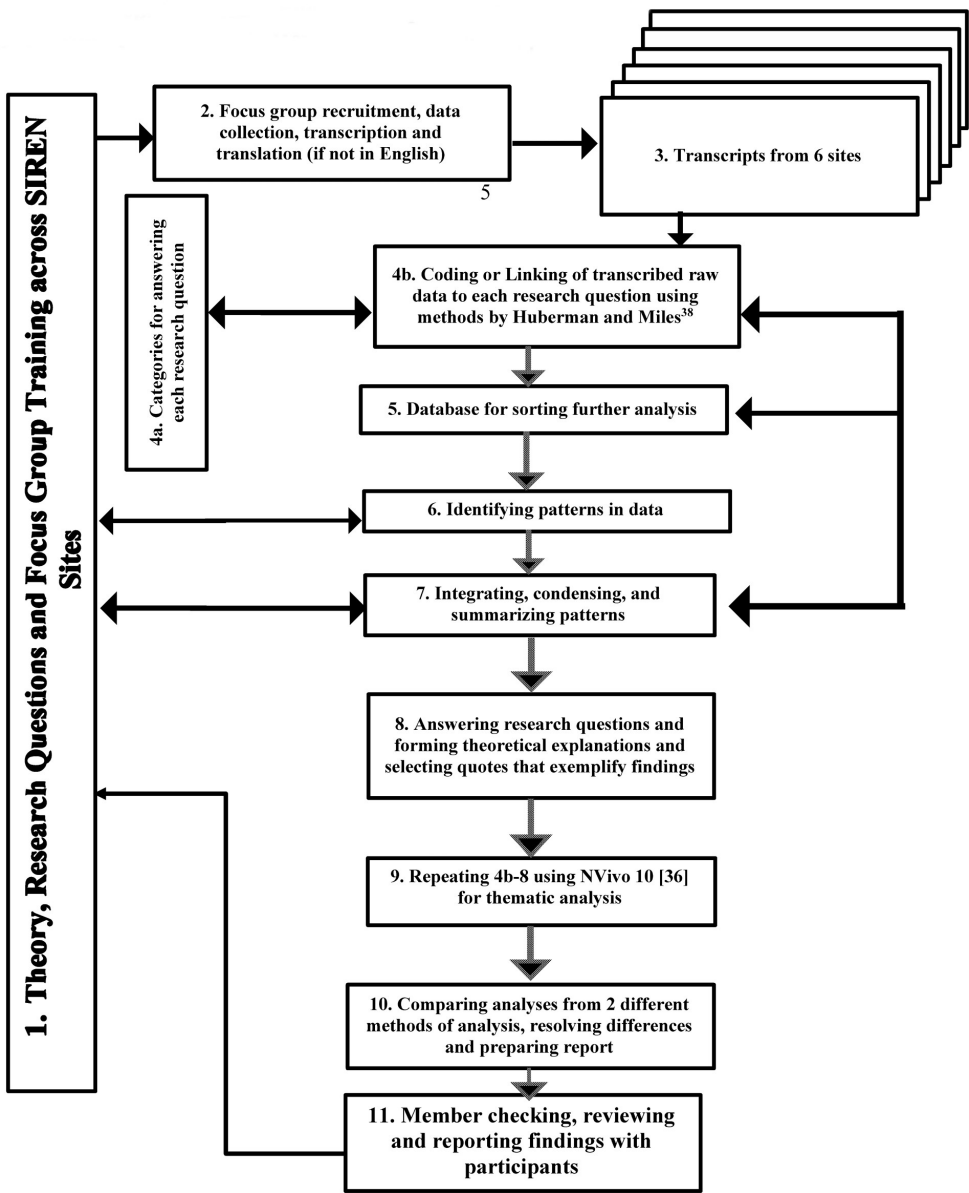
Overview of steps in the siren focus group process for reporting, analyzing and reporting findings [37].

Additionally, NVivo 10 software [36] was used to re-analyze the data first by using Kleinman’s model [28] and each of the coded data segments was then coded into the appropriate level of the SEM [27]. The coded data segments from the first coding and then from NVivo coding were extracted, compared and differences resolved, condensed, and summarized with examples of major and minor themes (including diverse themes) [37, 38]. The themes and quotes were then provided for feedback from each of the SIREN sites and there were no disagreements when reviewed during monthly Skype meetings or during manuscript review by each of the authors. A summary of the steps for the methods and coding process is shown in Fig 2.

## Results

### Domain 3 findings

The characteristics of the FG and participants are shown in Table 1. The major and minor or divergent themes are presented using a condensed framework from Kleinman’s Explanatory Model of Illness [28] that includes categorization of: i) causes of stroke and why it started when it did; ii) chief problems, severity and duration of the problems caused by the stroke; iii) fears related to stroke; and iv) treatment. The direct quotes of the FG participants are italicized (with quotation marks for short quotes or indented for longer quotes). The results are also organized by levels of the SEM of i) individuals; ii) interpersonal/social relationships and living conditions; iii) organizations and communities, and iv) social and economic policies. We then examine the recommendations for working effectively with communities to increase participation in SIREN’s case-control study for exploring the genetic, genomic, and phenomic causes related to stroke.

### Causes of stroke and why it started when it did

The major theme across all groups for the cause of stroke at the individual level centered around “*stress*” [mentioned by 51 stroke survivors (SS), ages 34–80; 26 community leaders (CL), ages 22–80; and 32 health care workers (HCW), ages 26–64]. A multitude of other factors identified as contributing to stress included *“worry*,*” “lack of rest*,*” “spiritual battle/terrestrial powers”* (but not further explained), *“overloading the brain”* or *“not giving the mind a rest*,*” “bitterness*,*” “loneliness” and “fear*.*”* Physiological causes of stroke, the second most common theme, included “*high blood pressure*” (mentioned by 35 SS, ages 37–80; 26 CL, ages 30–78; and 43 HCW, ages 23–64) which was attributed to lifestyle factors such as *“eating heavy meals or high fat meals and going to sleep*,*” “smoking” “drinking alcohol*,*” “sedentary lifestyle” “coffee and caffeine*,*” “high blood cholesterol*, *diabetes*, *as well as genetics or our genes*.*”* Again, at the individual level, stress and high blood pressure were the major reasons for why a stroke started when it did. And one of the major reasons for high blood pressure contributing to stroke was *“not taking the medications”* (mentioned by 12 different SS, ages 32–72) or *“running out of medications*” (mentioned by 9 different SS, ages 32–80). Reasons for not taking the medications or running out of medications included *“These pills made me feel bad*.” (SS, age 52) or *“I did not have the money to get my medicine*.*”* (SS, age 48) or *“My sister had all sorts of problems from taking the pill and told me not to take them or I would be cursed too*.*”* (SS, age 49). The relatively common minor or divergent theme across most groups was that stroke and reasons for why it started was a *“curse”* (6 SS, age 48–78) either from *“evil spirits*” (2 SS, age 74–77) or *“evil imagination”* (1 SS, age 32) or a *“sin from God”* (3 SS, ages 36–80) or “*punishment from Allah”* (1SS, age 48).

The interpersonal/social relationships, living conditions, and communities that contributed to stress and physiological causes of stroke, particularly high blood pressure were diverse, but included “*western lifestyle*,” (2 CL, age 61–78) “*poverty and slum living*,” (CL, age 44; HCW, age 53) “*environmental pollutants*, *second hand smoke*, *and bad air*.” (CL, age 39) “*Civilization and modernization has made those of us*, *especially in urban areas prone to more stress*. *This one is driving a Jeep so I must have Jeep*. *There is a remote for everything*!*”* (CL, age 38) Another cause of stress, high blood pressure and stroke was family stress and dysfunction. One particular theme related to multiple wives for men, and women who are in a marriage where the man has multiple wives or practices polygamy. *“Unnecessary stress like when a man has four wives so they tend to have too much family issues to worry about*, *no rest*, *no food; all these can cause high blood pressure then hypertension*, *then stroke*.*”* (CL, age 58) Additionally, other groups identified the multiple wives of men as being at risk for stress, high blood pressure and stroke because of poverty and other stressors related to feeling abandoned if they did not perform “*wifely duties*.” (CL, age 48)

For the cause of stroke and why it happened when it did, no social and economic policies were specifically mentioned other that those above as living conditions in communities. However, none of the different sites or groups of participants (community and faith leaders, persons with stroke or health care providers) specifically attributed the cause of stroke to social or economic policies. The divergent *“curse*,*”* or *“evil”* or *“sin”* was also not attributed to social or economic policies. There were no major differences across sites or across groups related to causes of stroke and why the stroke happened at a particular time except health professionals in urban sites shared a more in-depth discussion of the physiological and scientifically based causes of stroke than other groups and the health professionals in rural sites shared more causes of stroke that are not clearly linked to scientific evidence to support the causes such as *“polygamy*,*”*(5 HCW, age 30–45) *“environmental factors such as when you sleep with the generator on consistently*, *it will lead to pressure and later hypertension*, *then later stroke*.”(2 HCW, ages 43–55) And another environmental effect was *“severe heat*.*”*(4 SS, ages 38–80) Additionally, as high blood pressure was shared as a cause of stroke across all except two groups, there was no mention of limiting salt or salty food intake to help with control of high or elevated blood pressure across all groups except for one of the health professional groups that included two nutritionists. One of the nutritionists only briefly added at the end of a long list of foods to avoid that “high salt intake is possibly linked to unhealthy eating due to the aggregate use of it for preserving foods.” However, “*high fat foods*” and “*unhealthy eating*” were discussed in several groups of stroke survivors (6 groups), community members (5 groups) and health professionals (7 groups).

### Chief problems, severity and duration of the problems caused by the stroke

All groups shared that stroke has a major effect on the individual, their interpersonal/social relationships and living conditions especially related to their ability to return to a normal life, including being a contributor to their family, organizations and their communities. Most groups shared that social and economic policies are needed. The major themes related to chief problems, their severity and duration centered on the experiences of stroke survivors and their caregivers, as well as interpersonal/social relationships, while the identified effects on organizations and communities, were minimally discussed across all groups. *“Stroke causes many problems including paralysis*, *problems swallowing and eating*, *talking and moving around*. *And the problems can last a short while or a lifetime*. *These problems affect the individual*, *their family*, *their income*, *and their abilities to become a productive member of our community*. *Our government needs to help with policies that support the families*.*”* (CL, age 54) There was no agreement on minor or divergent themes offered across groups.

### Fears related to stroke

The major and minor/divergent fears related to stroke were at the individual and interpersonal/social relationships, living conditions and organizational levels but none were attributed to communities or social and economic policies.

Fears were common and many expressed: *“death*, *redundancy*, *burden or dependence on family*, *stroke complications*, *and immobility*.*” (23 SS*, *ages 42–80) “Stigma*, *financial and economic bondage*, *but the greatest fear is having it (stroke) again*, *and immobility*.*”* (SS, age 42) Health professionals reported that *“some stroke survivors wish for death because they do not want to be a burden to family*.” (5 HCW, ages 23–56) A divergent but common theme across groups was *“Many fear that hospital workers and relatives may decide to abandon them—particularly females fear husbands will divorce or abandon them and they will be neglected*.” (HCW, age 39) Another divergent theme related to reasons the stroke survivor worries: *“I worry a lot*. *People think I have done something wrong and that (stroke) is my punishment*. *They don’t see it as a form of sickness*.*”* (SS, age 68)

### Treatment and results

*“The whole essence of admissions is to address the immediate complications of stroke and to hasten the control of early rehabilitation*. *And to facilitate full recovery if possible or to learn to live with it (stroke damage)*.*”* (HCW, age 43) The health care providers reported that *“We believe in healing of diseases*. *We do not believe in management of disease*.*”* (HCW, age 49) A minor common theme across the health professional groups is illustrated by the following two quotes: *“A lot of people have no idea what stroke is all about*, *actually most people*, *and it is high time we educate them about it to enable them to come to hospitals in time and not waste their time with the spiritualists or herbalists or traditionalists*.*”* (HCW, age 62) *“Lots of folk go to traditional healers or herbalists*, *chemist or quack*. *The pastor is called to come and pray before going to hospital*. *If we believe that this is not a medical but a spiritual illness*, *it is better to pray than to use the meds*.” (HCW, age 61) The stroke survivors frequently reported a delay in seeking care from hospitals and health care providers. Many reported seeking alternative “*treatment from their church and prayer from their pastor*,” (SS, age 57) as well as not recognizing the signs and symptoms of a stroke until several days or even weeks have passed and the stroke survivor or family members do not see improvement in symptoms.

The health professionals (HCW, age 63) also reported that *“Most patients and their families have lots of questions after their stroke*: *Can I walk*, *drive*, *cook*, *and perform conjugal duties with my wife*? *So gently we nurse them in the right direction—physiotherapy*, *eat with family but with different foods—more healthy foods”* while other health professionals reported:

*You know patient counseling is not done in our clinics and it is not good—We need to educate our patients about their conditions and how to prevent some of the complications*. *People come in and say ‘when my drug got finished I thought that was the end of it*.*’ ‘I got better so I thought I don’t need these medications anymore*.*’* (HCW, age 55)

Another major theme was *“Lots of care—we frequently turn him*, *feed him*, *bed sores may increase the task of managing him and we have insufficient manpower and equipment to manage*.*”* (HCW, age 64) A stroke survivor (age 47) added *“and the government should help cushion financial implications*, *cost of drugs*. *The drugs are expensive—do something to help us”* while another (SS, age 47) stated *“I think the government or ministry of health should reserve some money for research in this area so we can deal with this epidemic……*.*”*

### Working effectively with communities to increase participation in SIREN’s case-control study for exploring the genetic, genomic, and phenomic causes related to stroke

At the individual level, it was clear: *“People want rewards for their time and for genetic testing to be successful*, *it should be confidential so he/she will not be denied anything in the future such as a job*.*”* (SS, age 38) Thus, social and economic policies are needed to protect the individual so he or she can maintain privacy with genetic testing.

*“Well*, *I think the team should be well-guided how they will present themselves to the community because in Yoruba land if you know how to talk*, *you should as well remember those that know how to interpret it*. *But if you train then go home*, *we go home and give awareness*. *We talk to them and then we choose time that will come to us*.*”* (CL, age 48) A minor theme related to the collection of blood: *“You know we cherish our blood and so many myths that surround the issue of blood collection in our environment*. *Be cognizant of that angle and know how to diffuse the myths and all the impressions*. *Properly educate as they say we should run away from blood—the issue of ebola*.*”* (SS, age 56) Another minor theme was: *“And government things always have a starting but finishing is the problem*. *Involve media people both print and the television so we hear it over and over*. *Make it not new to them*. *And we hope to have the results of the report of the research at the end of the day that will give us sense of belonging*, *at least we participated…*..*we can continue to move among our people*. *This research must not be allowed to die*. *If we reach out to this kind of people who are very close to the community*, *the sky will be the limit*. *God is going to help us*.*”* (HCW, age 34)

Overall, quotes from one of the participants in the health care provider groups seemed to sum up our findings related to effects of stroke across the SEM:

*It (stroke) is the spectrum from mild to severe affecting the patient himself*, *the family*, *the hospital*. *The patient becomes dependent and may be exhausted of resources*. *The family can become exhausted financially*, *psychologically*, *physically and emotionally because attention will be required…*.*and the availability of such manpower might not be there*. *To the hospital*, *it increases hospital stay and attention*, *and gradually little interest might be given to such patient*. *(After discharge) the stroke patient needs to be visited even at home*. *We lack facilities and manpower to follow up the patient in his own environment and see how he/she is coping with his/her disability and proper rehabilitation*. (HCW, age 52)

And stroke survivors and community and faith-based leaders summarized how to work effectively with organizations and communities related to the SIREN recruitment, as well as adding some advice related to social, economic and government policies related to stroke:

*You know this is political days and I know the test you may want to do may involve blood*. *So my concern is that people are now weary of things that may lead to whether they want to take any part of their body for anything*. *So you really need good advocacy before that type of test can take place*. *Tread softly*. *Develop good rapport and…*.*have resources to carry out the thing effectively and efficiently*. *You must go through community and religious leaders*. *They (*the community members) *believe our spiritual head cannot mislead us*. (CL, age 47)

*You need to sensitize the public*. *We have lots of strategies you could embrace—maybe you come on Google with jingles*, *or drama sketches*, *you come on play*. *Let’s even have street campaigns provided you have some drummers and some t-shirts so you can create awareness among the populace……Educate the community*, *use media*, *TV*, *radio*, *and churches and mosques to get the word out*. *Use volunteers to reach communities*, *go to houses*, *share leaflets*, *and read to those who cannot read……Research needs to be disseminated or people claim they are used for institutions to make profit*. *Overall*, *the major concern goes to government at all levels*. *There is a need for them to pay more attention to health care delivery and to research*. (CL, age 62)

## Discussion and conclusions

This report documented knowledge, attitudes, perception of risk factors for developing stroke, health seeking behavior related to stroke care, and willingness to be part of the genomic study. These findings involved stroke survivors and their families (n = 58), health care staff (n = 56), and key opinion leaders in the community (n = 51) with a total of 165 informants, which was a very large sample size for a qualitative study.

### Triangulation of FG findings with SIREN survey and SIREN case control study

When the qualitative data from this study are triangulated with phenomics quantitative survey data (defined as the measurement of phenomes—the physical and biochemical traits of participants with stroke and community controls—as they change in response to genetic mutation and environment influences), we found similarities as well as differences in community knowledge, attitudes, and perceptions related to stroke. If the FGS findings had been available when the survey was designed, the findings could have been validated by additional questions in the survey. However, the data were collected during the same period of time. The SIREN survey results thus far [Personal communication from Rufus Akinyemi related to knowledge, attitudes and practices of West Africans on genetic studies of stroke: evidence from the SIREN study] included 4,632 stroke survivors and controls (those who have not had a stroke) recruited into the SIREN study in Ghana and Nigeria. The following risk factors of stroke were reported by the participants: high blood pressure (59.2%), stress (39.8%), alcohol (28.4%), bad diet (28%), diabetes mellitus (24.8%), smoking (24.1%), high cholesterol (23.1%), obesity (21.8%), family history (15.5%), genetics (12.5%), old age (12.2%), evil spirit/witchcraft (8%) and will of God (4%). A report of further analysis of 2118 case-control pairs in the SIREN study identified the modifiable risk factors for stroke in Ghana and Nigeria (listed in descending order) as hypertension (90.8%), dyslipidemia (35.8%), regular meat consumption (31.1%), elevated waist-hip ratio (26.5%), diabetes (22.1%), low green leafy vegetable consumption (18.2%), stress (11.6%), added salt at the table (5.3%), cardiac disease (4.3%), physical inactivity (2.4%), and current cigarette smoking (2.3%) [39].

Our FG participants of 58 stroke survivors and 51 community leaders similarly reported all of the above causes (but no emphasis on salt or salty foods). The major themes in the FG analysis were that stress and high blood pressure were major causes of stroke, and they expressed that stress was the reason that the stroke happened when it did. Neither of the quantitative surveys capture “why stroke started when it did,” and also do not capture recommendations from community leaders or stroke survivors for specific actions to improve prevention of stroke or recommendations for stroke recovery and participation in genomic research. This information is captured from this qualitative study. It is crucial to remember that for interventions to be successful, we need to explore cultural relevance from the perspectives of the community members and qualitative explorations can provide that input. When the survey participants were asked about intended action steps to be taken should a relation or relative develop a stroke, they reported the following actions: go to hospital (81.9%), seek a specialist doctor (12.3%) and less than 1% would seek spiritual help or call a herbalist; yet seeking alternative care and delaying care from hospitals and doctors was a common theme in the FG. Another study in Nigeria (unrelated to SIREN) reported that only 28.9% of the patients with stroke presented to accident and emergency within the first 6 hours of onset of stroke [40].

When little information is known about a population’s beliefs, attitudes and practices, qualitative research may be considered essential to developing more insights into the development of quantitative measures. If the SIREN researchers had the qualitative data from the FG prior to developing the survey, we could have added additional questions that would have provided more insights into prevention and management of stroke, especially related to delays in seeking hospital care for stroke and for medication management for high blood pressure.

### Limitations and learning from other studies

In SIREN, it is important to note that all of the stroke patients who participated in the survey, as well as some of the controls, were recruited from hospitals and clinics so there is a possibility that they were biased towards seeking care from hospital or specialist doctor and the SIREN survey did not capture a delayed entry into hospitals and clinics.

There are other limitations to the current FGs and qualitative analyses that prevent us from deeper exploration of community attitudes, beliefs and practices related to stroke prevention and recovery. Specifically, the influence of religious beliefs across various groups of participants is not explored. It is important to note that in a study conducted in urban and rural communities in Tanzania, specifically in Dar-es-Salaam, the commercial capital of Tanzania, where Muslims make up 70% of the residents, stroke was “believed to emanate from supernatural causes (demons and witchcraft) while in rural Hai, explanation drew mostly on ‘natural causes (hypertension, fatty foods, stress) [41].” Those communities with beliefs of natural causes first chose hospital treatment while in Dar many avoided the hospital and used natural healers. A key finding was that causation beliefs shaped treatment choices. Thus, a clear understanding of each of our communities rather than “grouping” all the SIREN communities together is critical for community focused interventions. Also, if the budget was larger, rather than FGs, individual qualitative interviews could have been conducted and may have provided a more in-depth understanding of the attitudes, beliefs, and practices. A larger number of individual interviews are usually required to reach data saturation, and thus a larger budget for conducting the interviews and analyzing the additional data generated by the larger sample size. The FGs captured group-think or group discussion and allowed for ongoing validation of data within each group. To further address this limitation of our current qualitative and quantitative studies in SIREN (and other future studies), we need to further explore and integrate local beliefs, attitudes and practices into improving stroke prevention and recovery. One specific example that needs further research is the role of diet and specific foods or food additives such as salt or sodium in high blood pressure and stroke prevention, particularly since salt is used to preserve many foods in the two countries. Probes that encouraged further discussion related to diet would have offered additional insights into the role of particular foods or food additives in blood pressure control.

We can also learn from another qualitative study examining diabetes in urban and rural Ghana [42]. The author reported that i) “cure seeking constitute healer shopping between biomedical, ethnomedicine, and faith healing” and ii) “medical inaction constituted passive disengagement from medical management [42].” Also, related to the fears of stroke in our study, women expressed some concerns about fear of abandonment. Wives feared that husband would leave them and no such fears were expressed among husbands. In epilepsy similar findings were reported in studies on sociocultural issues where females were more likely to be abandoned and face more stigma than males [43, 44]. However, several studies in Nigeria [45, 46] emphasized the critical need for patient and family education related to the causes of stroke, effects of drugs and goals for a successful recovery, but the studies reported there is little time for physicians and staff to address these needs [45]. The role of poverty and the low capacity of the health care system to manage stroke also affects care. Thus, “patients and their families can leave clinic feeling ill-informed [45],” and often turn to God as a source of hope and recovery [46].

Patients with stroke may also have other issues and care must be individualized. A study in Ghana reported that approximately 80% of patients with stroke experience mild-to moderate degree of stigma related to their stroke and these stigmatized individuals were more likely to be depressed and have lower levels of quality of life [47]. Although not an identified major or minor theme from our FGs, the stigma, depression, and lower quality of life associated with stroke was mentioned in several FGs and implied in many. Additionally, Baatiema and colleagues [48] examined barriers to evidence-based acute stroke care explicated by health professionals in Ghana and concluded that we need to “consider the contrasts and uniqueness” of community-based barriers in designing quality improvement interventions to optimize patient outcomes related to stroke.

### Strengths and future directions

This qualitative approach of SIREN provides a model for other high-risk stroke populations around the world to explore the views of stroke survivors, their families, community members, health providers and researchers, and to use the findings from SIREN and other studies to improve health. These new perspectives can also be used to raise new questions and hypotheses about stress and stroke, as well as barriers to blood pressure control. Additionally, the findings and comparisons have the potential for changing both policy and practice related to stroke prevention, management and recovery, and genetic research in SSA.

After a systematic search of PubMed, Medline, Google Scholar, and review of published research related to stroke in Africa, to our knowledge, this is the first research that qualitatively explores and contrasts knowledge, attitudes/beliefs and practices of stroke survivors and their families, community and faith-based leaders, and health professionals across multiple communities in both Ghana and Nigeria in SSA. Also, this is the first qualitative study to specifically explore community willingness to participate in genomic research focused on stroke and to obtain suggestions from patients, family, community leaders and health care providers for recruiting persons to participate in the genomic and phenomic stroke research. These findings certainly add to both prior qualitative studies and current quantitative data from SIREN surveys that have been done and reported during the same period of time. Additionally, these findings can help to explore and further develop culturally sensitive and acceptable community-based educational interventions towards improving acceptability of genomic studies and the reduction of the stroke burden across SSA.

Future plans include further exploring and integrating local beliefs, attitudes and practices as well as using our current findings to improve stroke prevention and recovery, and community participation in research. We will continue to identify effective methods for disseminating SIREN findings across the participating communities and work with the SIREN Community Advisory Boards to develop and implement policies to support improving stroke care and outcomes. Our goal continues to focus on reducing the tremendous burden of stroke for patients, their families, and communities through research and partnerships to improve practice and policy.

## References

[1] Feigin VL, Krishnamurthi RV, Parmar P, Norrving B, Mensah GA, Bennett DA, et al Update on the global burden of ischemic and hemorrhagic stroke in 1990–2013: The GBD 2013 study. Neuroepidemiology. 2015;45(3):161–76. 10.1159/000441085 26505981 10.1159/000441085 PMC4633282

[2] Owolabi MO, Akarolo-Anthony S, Akinyemi R, Arnett D, Gebregziabher M, Jenkins C, et al The burden of stroke in Africa: a glance at the present and a glimpse into the future: review article. Cardiovascular J Africa. 2015;26(2):S27–S38. 10.5830/cvja-2015-038 25962945 10.5830/CVJA-2015-038 PMC4557491

[3] Taming the burgeoning stroke epidemic in Africa: stroke quadrangle to the rescue (P01.002). Neurology. 2012;78(Meeting Abstracts 1):P01.002-P01. 10.1186/1471-2377-12-78 22097671

[4] Ovbiagele B. National sex-specific trends in hospital-based stroke rates. J Stroke and Cerebrovascular Dis. 2011;20(6):537–40. 10.1016/j.jstrokecerebrovasdis.2010.03.007 20719540 10.1016/j.jstrokecerebrovasdis.2010.03.007

[5] Arisegi SA, Awosan KJ, Oche MO, Sabir AA, Ibrahim MT. Knowledge and practices related to stroke prevention among hypertensive and diabetic patients attending specialist hospital, Sokoto, Nigeria. Pan African Med J. 2018;29 doi: 10.11604/pamj.2018.29.63.13252 29875944 10.11604/pamj.2018.29.63.13252 PMC5987157

[6] O'Donnell MJ, Xavier D, Liu L, Zhang H, Chin SL, Rao-Melacini P, et al Risk factors for ischaemic and intracerebral haemorrhagic stroke in 22 countries (the INTERSTROKE study): a case-control study. Lancet. 2010;376(9735):112–23. 10.1016/S0140-6736(10)60834-3 20561675 10.1016/S0140-6736(10)60834-3

[7] Yusuf S, Rangarajan S, Teo K, Islam S, Li W, Liu L, et al Cardiovascular risk and events in 17 low-, middle-, and high-income countries. New England J Med. 2014;371(9):818–27. 10.1056/nejmoa1311890 25162888 10.1056/NEJMoa1311890

[8] Hall MJ, Levant S, DeFrances CJ. Hospitalization for stroke in US hospitals, 1989–2009. Diabetes. 18(23):23. 22617404

[9] Norrving B, Kissela B. The global burden of stroke and need for a continuum of care. Neurology. 2013;80(Issue 3, Supplement 2):S5–S12. 10.1212/WNL.0b013e3182762397 23319486 10.1212/WNL.0b013e3182762397 PMC12443346

[10] Wang Y, Rudd AG, Wolfe CDA. Age and ethnic disparities in incidence of stroke over time: the South London stroke register. Stroke. 2013;44(12):3298–304. 10.1161/STROKEAHA.113.002604 24114452 10.1161/STROKEAHA.113.002604

[11] An SJ, Kim TJ, Yoon B-W. Epidemiology, risk factors, and clinical features of intracerebral hemorrhage: an update. J Stroke. 2017;19(1):3–10. 10.5853/jos.2016.00864 28178408 10.5853/jos.2016.00864 PMC5307940

[12] Yan LL, Li C, Chen J, Miranda JJ, Luo R, Bettger J, et al Prevention, management, and rehabilitation of stroke in low- and middle-income countries. eNeurologicalSci. 2016;2:21–30. 10.1016/j.ensci.2016.02.011 29473058 10.1016/j.ensci.2016.02.011 PMC5818135

[13] Kernan WN, Ovbiagele B, Black HR, Bravata DM, Chimowitz MI, Ezekowitz MD, et al Guidelines for the prevention of stroke in patients with stroke and transient ischemic attack: a guideline for healthcare professionals from the American Heart Association/American Stroke Association. Stroke. 2014:STR. 0000000000000024. 10.1161/STR.0000000000000024 24788967

[14] Jenkins C, Arulogun OS, Singh A, Mande AT, Ajayi E, Calys-Tagoe B, et al Stroke investigative research and education network. Health Education & Behavior. 2016;43(1_suppl):82S–92S. 10.1177/1090198116634082 27037152 10.1177/1090198116634082 PMC4905563

[15] Akpalu A, Sarfo FS, Ovbiagele B, Akinyemi R, Gebregziabher M, Obiako R, et al Phenotyping stroke in Sub-Saharan Africa: stroke investigative research and education network (SIREN) phenomics protocol. Neuroepidemiology. 2015;45(2):73–82. 10.1159/000437372 26304844 10.1159/000437372 PMC4604029

[16] Akinyemi RO, Ovbiagele B, Akpalu A, Jenkins C, Sagoe K, Owolabi L, et al Stroke genomics in people of African ancestry: charting new paths: review article. Cardiovascular J Africa. 2015;26(2):S39–S49. 10.5830/cvja-2015-039 25962947 10.5830/CVJA-2015-039 PMC4557488

[17] Stroke investigative research & educational network (SIREN) 2013 [cited 2018 May 6, 2018]. Available from: https://h3africa.org/consortium/projects/16-projects/82-stroke-investigative-research-educational-network-siren.

[18] High-level principles on ethics, governance and resource sharing. 2013 [cited 2018 April 2]. Available from: https://h3africa.org/about/ethics-and-governance.

[19] Sandelowski M. What's in a name? qualitative description revisited. Research in Nurs & Health. 2009:n/a-n/a. 10.1002/nur.20362 20014004 10.1002/nur.20362

[20] Sandelowski M. Focus on research methods-whatever happened to qualitative description? Research in Nurs & Health. 2000;23(4):334–40. 10.1002/1098-240x(200008)23:4<334::aid-nur9>3.0.co;2-g 10940958

[21] Consolidated criteria for reporting qualitative research (COREQ): a 32-item checklist for interviews and focus groups. International J for Quality in Health Care. 2007;19(6):349–57. 10.1093/intqhc/mzm042 17872937 10.1093/intqhc/mzm042

[22] O’Brien BC, Harris IB, Beckman TJ, Reed DA, Cook DA. Standards for reporting qualitative research. Academic Med. 2014;89(9):1245–51. 10.1097/acm.0000000000000388 24979285 10.1097/ACM.0000000000000388

[23] Elliott R, Fischer CT, Rennie DL. Evolving guidelines for publication of qualitative research studies in psychology and related fields. British J Clinical Psychology. 1999;38(3):215–29. 10.1348/014466599162782 10.1348/014466599162782 10532145

[24] Mauthner NS, Doucet A. Reflexive accounts and accounts of reflexivity in qualitative data analysis. Sociology. 2003;37(3):413–31. 10.1177/00380385030373002

[25] Krueger R, Casey M. Focus groups: a practical guide to applied science Thousand Oaks, CA: Sage; 2009.

[26] Tremblay MA. The key informant technique: a nonethnographic application. American Anthropologist. 1957;59(4):688–701.

[27] Promoting health: intervention strategies from social and behavioral research. American J Health Promotion. 2001;15(3):149–66. 10.4278/0890-1171-15.3.149 11265579 10.4278/0890-1171-15.3.149

[28] Culture, illness, and care. Annals of Internal Medicine. 1978;88(2):251 10.7326/0003-4819-88-2-251 626456 10.7326/0003-4819-88-2-251

[29] Beaton D, Bombardier C, Guillemin F, Ferraz MB. Recommendations for the cross-cultural adaptation of health status measures New York: American Academy of Orthopaedic Surgeons 2002:1–9.

[30] Beaton DE, Bombardier C, Guillemin F, Ferraz MB. Guidelines for the process of cross-cultural adaptation of self-report measures. Spine. 2000;25(24):3186–91. 10.1097/00007632-200012150-00014 11124735 10.1097/00007632-200012150-00014

[31] Clarke V, Braun V. Thematic analysis Encyclopedia of Quality of Life and Well-Being Research: Springer Netherlands; 2014 p. 6626–8.

[32] Braun V, Clarke V. Using thematic analysis in psychology. Qualitative Res in Psychology. 2006;3(2):77–101. 10.1191/1478088706qp063oa

[33] Engle M. Qualitative data analysis: an expanded sourcebook (2nd Ed.) Matthew B. Miles and A. Michael Huberman. Thousand Oaks, CA: Sage publications, 1994, 336 pp. American J Evaluation. 1999;20(1):159–60. 10.1016/s1098-2140(99)80125-8

[34] Quinn Patton M. Qualitative research and evaluation methods. Sage; 2002.

[35] Simplifying qualitative data analysis using general purpose software tools. Field Methods. 2004;16(1):85–108. 10.1177/1525822x03259227

[36] Castleberry A. NVivo 10 [software program]. Version 10. QSR International; 2012. American J Pharmaceutical Education. 2014;78(1):25 10.5688/ajpe78125

[37] Gläser J, Laudel G, editors. Life with and without coding: two methods for early-stage data analysis in qualitative research aiming at causal explanations. Forum Qualitative Sozialforschung/Forum: Qualitative Social Res; 2013.

[38] Miles MB, Huberman AM, Saldana J. Qualitative data analysis: Sage; 2013.

[39] Owolabi MO, Sarfo F, Akinyemi R, Gebregziabher M, Akpa O, Akpalu A, et al Dominant modifiable risk factors for stroke in Ghana and Nigeria (SIREN): a case-control study. Lancet Global Health. 2018;6(4):e436–e46. 10.1016/S2214-109X(18)30002-0 29496511 10.1016/S2214-109X(18)30002-0 PMC5906101

[40] Owolabi L, Nagoda M. Stroke in developing countries: experience at Kano, Northwestern Nigeria. Stroke. 2012;7(1).

[41] Urban-rural contrasts in explanatory models and treatment-seeking behaviours for stroke in Tanzania. J Biosocial Science. 2007;40(01). 10.1017/s0021932007002295 17767790 10.1017/S0021932007002295

[42] Healer shopping in Africa: new evidence from rural-urban qualitative study of Ghanaian diabetes experiences. BMJ. 2005;331(7519):737 10.1136/bmj.331.7519.737 16195290 10.1136/bmj.331.7519.737 PMC1239976

[43] Birbeck GL, Chomba E, Atadzhanov M, Mbewe E, Haworth A. Women’s experiences living with epilepsy in Zambia. American J Tropical Med & Hygiene. 2008;79(2):168–72. PMC2556284 18689619

[44] The social and economic impacts of epilepsy on women in Nigeria. Epilepsy & Behavior. 2012;24(1):97–101. 10.1016/j.yebeh.2011.11.019 22445872 10.1016/j.yebeh.2011.11.019

[45] Hurst S, Arulogun O, Owolabi MO, Akinyemi RO, Uvere E, Warth S, et al The use of qualitative methods in developing implementation strategies in prevention research for stroke survivors in Nigeria. J Clinical Hypertension. 2016;18(10):1015–21. 10.1111/jch.12817 PMC8031481 27038071

[46] A study of perceived factors affecting patients' participation in outpatient stroke physiotherapy exercise in Nigeria. International J Therapy and Rehabilitation. 2012;19(10):581–90. doi: 10.12968/ijtr.2012.19.10.581

[47] Stroke-related stigma among West Africans: patterns and predictors. Neurological Sciences. 2017;375:270–4. 10.1016/j.jns.2017.02.018 28320146 10.1016/j.jns.2017.02.018 PMC5364027

[48] Baatiema L, de-Graft Aikins A, Sav A, Mnatzaganian G, Chan CKY, Somerset S. Barriers to evidence-based acute stroke care in Ghana: a qualitative study on the perspectives of stroke care professionals. BMJ Open. 2017;7(4):e015385 10.1136/bmjopen-2016-015385 28450468 10.1136/bmjopen-2016-015385 PMC5719663

